# Assessment of Mastitis Patterns in Serbian Dairy Cows: Blood Serum Metabolic Profile and Milk Composition Parameters

**DOI:** 10.3390/pathogens12111349

**Published:** 2023-11-14

**Authors:** Jovan Stanojević, Mario Kreszinger, Miodrag Radinović, Nebojša Kladar, Dragana Tomanić, Zoran Ružić, Zorana Kovačević

**Affiliations:** 1 Department of Veterinary Medicine, Faculty of Agriculture, University of Novi Sad, Trg Dositeja Obradovica 8, 21000 Novi Sad, Serbia; jovan.stanojevic@polj.edu.rs (J.S.); miodrag.radinovic@polj.uns.ac.rs (M.R.); tomanicd@live.com (D.T.); ruzicvet@gmail.com (Z.R.); zorana.kovacevic@polj.edu.rs (Z.K.); 2 Clinic for Surgery, Orthopaedics and Ophthalmology, Faculty of Veterinary Medicine, University of Zagreb, 10000 Zagreb, Croatia; 3 Center for Medical and Pharmaceutical Investigations and Quality Control, Faculty of Medicine, University of Novi Sad, Hajduk Veljkova 3, 21000 Novi Sad, Serbia; nebojsa.kladar@mf.uns.ac.rs; 4 Department of Pharmacy, Faculty of Medicine, University of Novi Sad, Hajduk Veljkova 3, 21000 Novi Sad, Serbia

**Keywords:** blood parameters, dairy cows, mastitis, metabolic profile, Serbia

## Abstract

Mastitis is one of the most important diseases in dairy cows, leading to substantial economic losses associated with decreased milk production and quality. Early detection of changes in metabolic and milk parameters is crucial for maintaining animal welfare and milk quality. This study aimed to detect patterns in metabolic and milk composition parameters in Serbian dairy cows affected by mastitis. It also examined the relationship between these factors in cows with clinical and subclinical mastitis, as well as in healthy cows. This study included 60 Holstein-Friesian cows with the same body score condition that were in the same lactation phase. They were divided into three groups of 20: clinical and subclinical mastitis and a control group of healthy cows. The categorization was based on clinical udder health and the California mastitis test. Blood serum metabolic profiles were measured using a Rayto spectrophotometer (Shenzhen, China), and milk composition was determined using MilcoScan^TM^ (Foss, Hilleroed, Denmark) and Fossomatic^TM^ (Foss, Hilleroed, Denmark) instruments. Significant increases in non-esterified fatty acids (NEFAs), beta-hydroxybutyrate (BHB), total protein, globulin, urea, total bilirubin, magnesium, and enzyme activity were noted in mastitis-affected cows compared to healthy ones. Additionally, mastitis-affected cows had higher total protein and globulin levels and increased somatic cell counts (SCCs), while albumin concentrations were decreased. Furthermore, a negative correlation between total protein and lactose suggested inflammation leading to reduced lactose levels due to cell damage, infection, and lactose use by mastitis pathogens. Hence, indicators of the energy and protein status of the metabolic profile, together with the chemical composition of milk, may be significant diagnostic tools for detecting, monitoring, and predicting the outcome of mastitis in cows.

## 1. Introduction

Bovine mastitis is one of the most important and common diseases in dairy cows, causing huge economic losses associated with reduced milk yield, increased therapy costs, and poor quality of milk and dairy products [1,2,3]. This disease represents inflammation of the mammary gland caused by physical trauma or microorganism infections [4]. Therefore, some authors suggested that decreased milk production accounts for approximately 70% of the total cost of mastitis [5]. The etiology of mastitis is continuously changing, with new microbial species being incriminated, and about 150 *bacterial* spp. have been isolated from bovine mastitic udders [6]. 

This pathology manifests itself in two forms, clinical and subclinical mastitis, according to the presentation of the clinical signs. The clinical manifestation involves noticeable changes in the milk and/or udder. In contrast, subclinical mastitis remains detectable solely through laboratory tests, without any visible signs of its presence [7,8]. 

Mammary glands have defense mechanisms against microorganisms that cause mastitis. These mechanisms are divided into innate and adapted immunity [9]. It is well known that neutrophils are important cells of the immune system. During infections of mammary glands, neutrophils are the first immune cells that migrate from the circulation to the inflammatory area. Furthermore, neutrophils require energy for their activity, and cows with a negative energy balance (NEB) have a predisposition for the occurrence of mastitis [10]. 

The cells of the immune system consume glucose as their first source of energy [11], but recent studies revealed that beta-hydroxybutirate (BHB) is also metabolized in response to immunological challenges [12]. Although the immune system can use ketone bodies as an energy source, cows with high serum concentrations of non-esterified fatty acids (NEFAs) postpartum had an increased incidence of mastitis [13]. When there are increases in the fatty acid and ketone concentrations in the body, alterations in respiratory burst activities occur, leading to a decrease in the viability of neutrophil leukocytes [14,15]. Therefore, the maintenance of the appropriate metabolic status is very important for mammary gland health. Furthermore, blood metabolite profiling is a valuable tool in veterinary medicine for assessing the health status of animals, helping veterinarians and farmers in making well-informed decisions to enhance animal health and productivity [16].

In addition to alterations in blood metabolic parameters, mastitis can also lead to changes in milk composition. These changes result from the migration of blood components into the milk due to a decrease in the integrity of the blood–milk barrier [12]. Furthermore, the functionality of this barrier is essential to enable milk secretion and to prevent the loss of blood components via milk in lactating animals [12]. As a consequence of damage to this barrier, milk components (e.g., lactose) may appear in the blood [17]. Therefore, the recovery of the blood–milk barrier after mastitis is crucial to regain milk quality [12]. Important indicators of barrier integrity include the somatic cell count (SCC), activity of lactate dehydrogenase (LDH), serum albumin (SA), and IgG in the milk [18,19]. 

The occurrence of mastitis can lead to different changes in the mammary gland that may vary based on the different breeds, treatment methods, and management practices; the presence of diverse microorganisms in the environment; and regional factors, such as geographical location [20]. Therefore, it is crucial to gain insight into the mastitis-causing pathogens in the mastitis control program, considering their specificity within the region. Additionally, the patterns of metabolic and milk composition parameters are crucial for the detection of mastitis in dairy herds as well as for assessing the functional activity of a mammary gland. Moreover, identifying udder health issues through the early detection of changes in metabolic and milk variables is vital for dairy farmers to ensure animal welfare and maintain milk quality and productivity [21]. 

Hence, the primary aim of this study was to determine if there was a pattern in the occurrence of mastitis among dairy cows due to changes in the serum metabolic profile and milk composition parameters. Furthermore, the relationship between the mentioned parameters in blood and milk was assessed in cows affected by clinical and subclinical mastitis, as well as healthy ones.

## 2. Materials and Methods

### 2.1. Sampling Procedure

This study was conducted at a dairy farm located in the Autonomous Province of Vojvodina, Republic of Serbia, during the spring of 2023. There were a total of 600 dairy cows on the farm. Altogether, 60 Holstein-Friesian cows with the same body score condition (3) and in the same lactation phase (between 90 and 120 days in milk) were included in this study. The cows were divided into three groups. The first group included cows with clinical mastitis, while in the second group included cows with subclinical mastitis. The third group included healthy cows. All groups were equal, with 20 cows each. The assessment of clinical mastitis relied on clinical examinations conducted by veterinarians at the farm, while the subclinical form of mastitis was verified using the California mastitis test, which indirectly measures the SCCs in milk samples. The clinical symptoms indicating udder inflammation included swelling, pain, and redness. These changes were used for the diagnosis of the clinical form of mastitis, along with the observable changes in the first jets of milk characterized by the presence of clots, color alterations, and density variations. Cows affected by the subclinical form of mastitis did not show any clinical symptoms of udder inflammation, and no observable changes were noticed in the first jets of milk. However, they did return positive (+) results in the California mastitis test (CMT), indicating the presence of the subclinical form of mastitis. In contrast, healthy cows showed no clinical changes in their udders or milk samples, and they tested negative in the CMT. 

#### 2.1.1. Milk Sampling and Analysis

Milk sampling was performed before the morning milking. Samples of milk were taken from the affected quarters of mammary glands in cows with clinical and subclinical mastitis, while in healthy cows milk sampling was performed from one quarter of the mammary gland. The first jets of milk were hand-milked and discarded. After that, the milk was hand-milked into a sterile bottle, and then the milk samples were transported to the Laboratory for Milk Hygiene at the Department of Veterinary Medicine, Faculty of Agriculture, University of Novi Sad. A chemical composition analysis was performed on a MilcoScan^TM^ instrument (Hilleroed, Denmark) and included the determination of milk fat, lactose, total protein in milk, casein, beta-hydroxybutyrate (BHB), acetone, urea, lactate dehydrogenase (LDH), and colony-forming units (CFUs). An analysis of the SCC was performed on a Fossomatic^TM^ apparatus (Foss, Denmark). 

#### 2.1.2. Blood Sampling and Analysis

Blood was sampled from the jugular vein after the morning milking. A tube with a clot activator for blood sampling was used, and the blood samples were transported to the laboratory within one hour. After 3 h of clotting at 4 °C and centrifugation (1500 G for 10 min), the blood serum was analyzed for the following biochemical parameters: glucose, non-esterified fatty acids (NEFAs), beta-hydroxybutyrate (BHB), triglycerides, total protein, albumin, globulin, urea, creatinine, total bilirubin, aspartate aminotransferase (AST), alanine transaminase (ALT), alkaline phosphatase (ALP), gamma-glutamyl transferase (GGT), lactate dehydrogenase (LDH), calcium (Ca), magnesium (Mg), and phosphorus (P), which were determined using colorimetric kits (Biosystem, Spain, and Randox, Carlisle, UK) and a Chemray spectrophotometer (Rayto, Shenzhen, China). All analyses were performed at the Laboratory of Pathophysiology, Department of Veterinary Medicine, University of Novi Sad. 

### 2.2. Statistical Analysis

**Figure 1:**
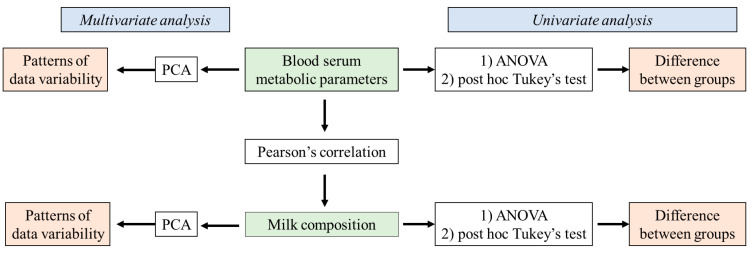
The scheme of data analysis.

The obtained results were summarized using Microsoft Office Excel (v2019) in the form of a matrix (size: 29 × 63) and processed using Statsoft Statistica (v12.5) (Tulsa, OK, USA). Data were analyzed by means of univariate and multivariate statistical methods (Figure 1).

The differences between the analyzed groups of animals (clinical mastitis, subclinical mastitis, and healthy animals) in terms of milk composition (milk fat, lactose, total protein in milk, casein, BHB, acetone, urea, LDH, CFUs, and SCC) and blood serum (glucose, NEFAs, BHB, triglycerides, total protein, albumin, globulin, urea, creatinine, total bilirubin, AST, ALT, ALP, GGT, LDH, calcium, magnesium, and phosphorus) parameters were estimated using a one-way ANOVA. In order to specifically define the groups that were contributing to the differences observed in the ANOVA, a post hoc Tukey’s test was applied. The differences were considered significant if *p* < 0.05.

The correlations between numerical continuous variables related to the milk composition and blood serum parameters (stated above) were estimated using Pearson’s correlation coefficient at a statistical significance of *p* = 0.05.

Furthermore, the variability of the evaluated data was assessed via the application of multivariate statistics using a principal component analysis (PCA) that was applied to datasets describing the blood biochemical parameters (glucose, NEFAs, BHB, triglycerides, total protein, albumin, globulin, urea, creatinine, total bilirubin, AST, ALT, ALP, GGT, LDH, calcium, magnesium, and phosphorus) and milk quality parameters (milk fat, lactose, total protein in milk, casein, BHB, acetone, urea, LDH, CFUs, and SCC). PCA is a dimension reduction technique enabling the description of initial dataset variability using a lower number of dimensions (principal components) that correlate with the original variables used to describe the samples’ variability. This approach enables better insight into the patterns of variability in the original data. 

## 3. Results

### 3.1. Metabolic Parameters in the Blood Serum of the Healthy Cows and Cows with Subclinical and Clinical Forms of Mastitis

The average concentrations of the metabolic parameters in cows with the clinical (1) and subclinical (2) forms of mastitis as well as healthy (3) ones are presented in Table 1. The table also illustrates the statistical differences (Supplementary Material Table S1) among these parameter values for the three groups of cows.

**Figure 2:**
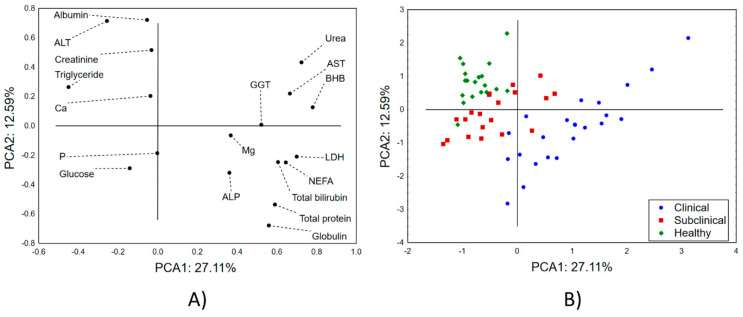
Principal component analysis (PCA) of blood parameters. The positions of the (**A**) variables and (**B**) samples (cows) in the space defined by the first two principal components.

The application of a principal component analysis (PCA) to the variables describing the biochemical parameters of the blood serum in the investigated groups of animals shows that the first two principal components describe almost 40% of the sample variability (Figure 2A and Supplementary Material Table S2). For the first principal component (PCA1), most of the variability is explained by the recorded levels of LDH, BHB, AST, urea, total bilirubin, and triglycerides. On the other hand, the shape of the sample variability is mostly correlated (in terms of the second principal component—PCA 2) with the recorded concentrations of albumin and globulin. The positions of the evaluated cows in the space defined by the first two principal components (Figure 2B) shows a predominant grouping of the cows with the clinical form of mastitis in the positive part of PCA 1 and the negative part of PCA 2, indicating higher levels of LDH, ALP, magnesium, GGT, NEFAs, total bilirubin, total protein, and globulin in these cows. On the other hand, healthy cows are mostly located in the negative part of PCA1 and the positive part of PCA 2, suggesting lower levels of the previously listed parameters in their blood serum and higher concentrations of albumin, ALT, creatinine, triglycerides, and calcium. Furthermore, it can be observed that the cows with the subclinical form are nested in the space between the healthy animals and the animals with the clinical form of mastitis, suggesting transitioning values for the previously mentioned blood biochemical parameters.

### 3.2. Milk Composition in Cows with Clinical and Subclinical Forms of Mastitis and Healthy Cows

Table 2 shows the average values of milk parameters in cows with the clinical form of mastitis (1) and the subclinical form of mastitis (2) and healthy (3) cows. The table also indicates the statistical differences (Supplementary Material Table S1) among these parameters in the three groups of cows.

**Figure 3:**
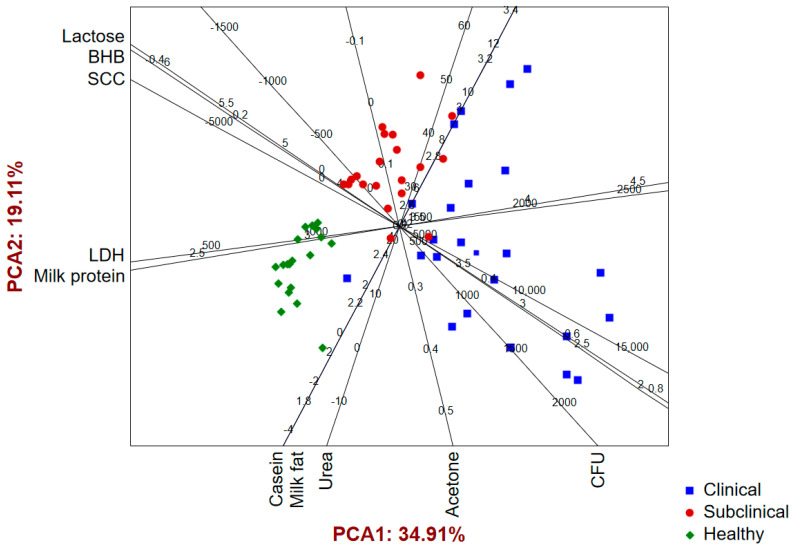
Principal component analysis biplot of milk parameters.

The application of a PCA to the results of the analysis of the milk samples obtained from the evaluated cows shows that the first two principal components describe around 55% of the sample variability (Supplementary Material Table S3). The PCA biplot (Figure 3) shows separate groupings of milk samples obtained from the three evaluated groups of cows. Milk samples obtained from cows with the clinical form of mastitis are mostly located in the positive space of PCA1 as a result of higher recorded results for BHB, SCC, CFUs, LDH, and milk protein, as well as lower concentrations of lactose. The milk samples of healthy cows and cows with the subclinical form of mastitis are mostly located in the negative part of PCA1 and are further separately grouped in terms of PCA2. Figure 3 shows that the lowest levels of LDH and protein concentrations are present in the milk of healthy cows, discriminating it from the other two groups of milk samples. In addition, in terms of the lactose and BHB concentrations, as well as the SCC, milk samples from healthy cows cannot be separated from milk samples obtained from cows with the subclinical form of mastitis.

### 3.3. Correlation between Metabolic Parameters of Blood and Chemical Composition of Milk

Table 3 shows correlations between the metabolic parameters of blood and the parameters of milk composition.

## 4. Discussion

Modern dairy farming often results in forced milk production, giving rise to metabolic disorders in cows. One of the most important diagnostic tools to predict such disorders and related subclinical diseases is the establishment of the physiological ranges of the biochemical parameters in a clinically healthy herd [22,23]. While microbiological analysis is undoubtedly essential to understanding the microbial etiology of mastitis, our study aimed to explore a complementary aspect of the condition. The study was designed to detect metabolic patterns associated with the occurrence of mastitis and examine the relationship between these factors in cows with clinical and subclinical mastitis, as well as healthy cows. Other authors also focused their research on this issue [24,25,26]. Microbiological analysis typically involves identifying and characterizing the bacteria causing mastitis, which is a different aspect of the disease. 

Moreover, mastitis is important and is the most common disease in dairy cows, leading to significant changes in the metabolic profile and milk composition. So far, when it comes to mastitis in Serbia, no one has focused on this issue yet. The relationships between milk and blood biochemical parameters and metabolic status in dairy cows during lactation were only determined in healthy cows in Serbia [27]. Taking into account the prevalence of mastitis and the importance of the dairy sector in Serbia, insights into the data from our study are important and valuable [28,29,30]. 

As a disorder of the mammary gland, mastitis leads to increased permeability of the blood vessels in an inflamed area and consequently affects the transition of metabolites and neutrophils from blood to milk [31,32]. Furthermore, different stages of metabolism can increase the prevalence of any disease; in particular, an NEB has an immunosuppressive effect on it. In the present study, the physiological ranges of the biochemical parameters were determined to predict the health status of the cows. The glycemia values of all cows included in this study (affected by the clinical and subclinical forms of mastitis and healthy cows) were lower than the physiological range of this parameter (2.5 to 4.2 mmol/L) [23] but did not show significant differences between the cows (affected with mastitis and healthy ones), which is in line with previous studies [33]. Interestingly, no statistically significant correlation was found between glucose and any other metabolic parameter in milk. Along with the concentration of glucose, the most important indicators of the energy balance are the NEFA and BHB concentrations in the blood serum. Increased values of NEFAs and BHB in the blood serum often arise in early lactation due to a decrease in feed intake when cows enter an NEB with a decrease in the glucose value [34]. Furthermore, mastitis may arise as a consequence of the NEB due to immunosuppression, but this disease can also lead to an NEB due to released cytokines that cause increased mobilization of NEFAs and consequently an increased BHB concentration in the blood serum [35,36]. On the other hand, a BHB decrease in the blood serum during the inflammation could be explained as a consequence of increased blood glucose, changes in the BHB supply via reduced rumen motility, an impairment of hepatic ketogenesis, or a combination of the aforementioned factors [37].

In the present study, the concentrations of NEFAs and BHB were significantly higher in cows with the subclinical and clinical forms of mastitis than in healthy ones. On the other hand, other research results showed that there was no difference in the level of BHB in the blood serum between herds with high and low mastitis incidences [13]. The researchers in the mentioned study assumed that the suggested difference in the immune function was not induced by BHB. In addition, our research results showed that the concentration of triglycerides in serum showed an opposite trend compared to the NEFA and BHB concentrations. Our results indicated that the cows with mastitis had a lower triglyceride concentration compared to the healthy cows. The reason for this could be that the excretory function of hepatocytes is reduced and the liver is not able to release triglycerides into the circulation. As a consequence, triglycerides accumulate in the liver, leading to a decrease in their concentration in the circulation [38]. Contrary to these findings, other researchers have found that there is no significant difference in the triglyceride values between cows affected by mastitis and healthy ones [39]. In the current study, the blood serum concentrations of NEFAs and BHB showed significant positive correlations with the milk fat, SCC, and LDH in the milk. It is well known that the SCC is increased in milk from cows with mastitis and represents a valuable diagnostic tool for detecting the subclinical form of mastitis [40]. Additionally, the LDH in milk is a sensitive indicator of epithelial cell damage. The LDH in milk originates from the damaged udder’s epithelial cells or the leukocytes in milk [41]. This could explain the results obtained in the present study, where significantly higher levels of SCCs and LDH were determined in the milk from cows with the subclinical and clinical forms of mastitis when compared to healthy ones. These results are in accordance with those from other authors [25,42]. Furthermore, the ability of ruminant mammary glands to produce milk components is determined by the number of cells secreting milk and their level of activity. Hence, the amount of milk produced and the concentrations of protein, lactose, and fat in the milk might be affected by the level of inflammation in the mammary gland [43], indicating that the decrease in the fat content during the intramammary infection occurs as a consequence of reduced synthetic and secretory capacities of milk alveoli and due to increased activity of the milk enzyme lipoprotein lipase. This is in accordance with studies by many other authors who recorded significantly lower fat concentrations in milk obtained from cows with mastitis than from healthy cows [44,45,46]. Interestingly, the present study found a significantly higher concentration of milk fat in cows with clinical mastitis compared to healthy ones. The explanation for this could be that increased lipomobilization, as a consequence, results in a higher NEFA concentration in cows with the clinical and subclinical forms of mastitis compared to healthy ones. Furthermore, the mammary gland uses NEFAs from the blood serum for the synthesis of milk fat [47]. This is a reason why the NEFA and BHB concentrations showed positive correlations with milk fat in the current study. On the other hand, triglycerides have shown significant negative correlations with milk fat, SCCs, and LDH. This relationship is linked to the liver’s functional activity, as it plays a significant role in the acute inflammatory response and the synthesis of acute-phase proteins in sick cows [48]. Moreover, a previous study demonstrated that mammary inflammation was associated with the impairment of liver metabolism and liver function [49]. In addition, the liver participates in the metabolism of NEFAs, which are released from peripheral adipose tissue. This increase in NEFAs in the circulation is a result of proinflammatory cytokines (tumor necrosis factor (TNF)-α and IL-1) released by macrophages during the infection [50]. Hence, due to the increased NEFAs in the circulation, the liver takes more NEFAs from circulation, converting them into triglycerides within the liver. The accumulation of triglycerides in hepatic cells and the inability of the liver to release them into the circulation causes their decrease [50].

During an udder infection, inflammatory leukocytes and damaged epithelial cells release different products such as hydrolytic and non-lysosomal enzymes, including LDH or β-galactosidase lysosomal enzyme, which adversely affect milk quality. Different milk enzymes, such as LDH and ALP originate from disintegrated blood leukocytes, interstitial cells, and damaged epithelial cells of the mammary parenchyma. These enzymes in the milk and blood serum of infected animals are considered to be the best biomarkers of udder health, as their levels increase during inflammation in mastitis-affected animals [33,51]. Kurjogi et al. [52] indicated a significant increase in the activity of ALP and LDH in the blood serum of cows affected by mastitis. Yehia et al. [53] recorded significantly higher concentrations of LDH, ALP, and GGT in the milk of cows affected by subclinical mastitis. Moreover, these authors recorded higher blood serum concentrations of LDH and ALP in cows with subclinical mastitis compared to healthy ones. Most of the cells contain LDH, and when these cells are lethally injured, the loss of membrane integrity can be assessed by monitoring the activity of LDH [54]. Our results indicate that the concentration of LDH in blood serum shows a positive correlation with the concentration of LDH in milk, as well as the total protein and SCC in milk, while a negative correlation was found with the lactose in milk. The significantly higher concentration of LDH in the blood serum in the cows affected by the clinical and subclinical forms of mastitis compared to healthy cows could be explained by the fact that destroyed secretory cells of the mammary gland release LDH [41]. 

 Creatinine is synthesized, linked to the absorption of creatine phosphate by the muscles, then released to the blood serum and cleared by the kidneys. In dairy cattle, this molecule has been found to be proportional to muscle activity, with specific reference to respiration rates and heart activity [55]. In the present study, a significantly higher concentration of creatinine was found in healthy cows compared to cows with the subclinical and clinical forms of mastitis. It is well known that muscle creatinine is the main source of plasma and urinary creatinine and that any change in the skeletal muscle mass in healthy hydrated animals with normal renal function results in a plasma creatinine concentration change [56,57].

On the other hand, the concentration of total bilirubin shows a significantly higher concentration in cows with the clinical form of mastitis than in those affected by the subclinical form and healthy cows. This probably occurs due to the reduced functional activity of the liver [58]. In addition, total bilirubin shows significant correlations with all examined parameters (milk fat, total protein, SCC, lactose, urea, BHB, and LDH) except the CFUs, casein, and acetone in the milk.

The differences in the concentrations of calcium and phosphorus were not statistically significant between these three groups of cows, but the concentration of magnesium was significantly higher in cows with the subclinical and clinical forms of mastitis compared to those who were healthy. Contrary to our findings, other researchers recorded that there was no significant difference in the magnesium concentration between cows affected by mastitis and healthy ones [59]. Therefore, the concentration of magnesium in blood serum showed significant positive correlations with the total protein and LDH in milk in the present study. This means that the blood concentration of magnesium increases together with some other specific parameters for mastitis in milk, such as LDH. The results of the current study confirmed that the blood and milk parameters are different in cows with the clinical and subclinical forms of mastitis and healthy cows. Specifically, higher concentrations of LDH, ALP, magnesium, GGT, NEFAs, total bilirubin, total protein, and globulin were present in the blood serum of cows with the clinical form of mastitis, which were not observed in healthy cows. This indicates that mastitis causes these changes. Das et al. [60] reported significant increases in the levels of total protein and calcium in cows affected by mastitis compared to healthy cows. On the other hand, these authors reported no significant differences in the concentrations of magnesium and ALT between sick and healthy cows [60]. Furthermore, these authors suggested that changes in hemato-biochemical parameters can be used as important indicators of the physiological or pathological state (mastitis) of the animal [60]. Furthermore, Saleh et al. [61] recorded significantly lower serum concentrations of total protein, albumin, and globulin in cows affected by subclinical mastitis compared to healthy cows. 

On the other hand, higher concentrations of albumin, ALT, creatinine, triglycerides, and calcium were recorded in the blood serum of healthy cows in comparison to cows affected by mastitis. The results obtained in the present study indicated significant differences in the milk composition of the examined parameters between cows affected by mastitis and healthy ones. Our research results indicated higher concentrations of BHB, SCC, CFUs, LDH, and total milk protein in cows with the clinical form of mastitis. Matei et al. [62] reported a significantly higher concentration of total protein in milk from cows affected by mastitis compared to healthy ones. Thus, this increase in the concentration of total proteins in the milk of cows affected by mastitis could be a consequence of the high permeability of blood vessels and the release of serum proteins from the blood into the milk, as well as the migration of leukocytes [63]. However, Qayyum et al. [51] found a lower concentration of total protein in milk from cows with mastitis compared to healthy ones. Furthermore, Bochniarz et al. [44] recorded significant decreases in the concentrations of total protein, lactose, and milk fat in cows with clinical and subclinical mastitis compared to healthy cows. Interestingly, in the present study, no significant differences in the concentrations of lactose and CFUs were found between cows affected by the subclinical form of mastitis and healthy cows.

Our study also showed a lower concentration of lactose in cows with the clinical form of mastitis than in healthy ones, which is consistent with the results from other authors [64]. The decreased concentration of lactose in the milk occurs due to damage in secretory cells caused by inflammation and infection and the use of lactose as a substrate for growing mastitis pathogens [65]. Therefore, for the diagnosis of subclinical and clinical mastitis, significant diagnostic tools might include the concentration of LDH in milk and total milk protein. 

## 5. Conclusions

Mastitis leads to significant changes in the metabolic profiles of dairy cows. These changes depend on the degree of damage to the mammary gland. The most important changes in the metabolic profiles of cows affected by mastitis are related to the energy status (NEFAs and BHB), the protein status (total protein, albumin, and globulin), and enzyme activity. These changes are related to changes in the chemical composition of milk. Hence, the comparative determination of the metabolic profile and the chemical composition of milk can be a good diagnostic tool for detecting, monitoring, and predicting the outcome of mastitis in cows. In addition, based on the metabolic profile and chemical composition of milk, it is possible to categorize a cow herd into groups with mastitis and healthy cows. The most important parameters in milk for diagnosing subclinical and clinical mastitis are the concentrations of LDH and total milk protein, as well as the concentrations of albumin and globulin in blood serum. Furthermore, it was noticed that mastitis induces elevated ketogenesis through increased BHB and NEFA concentrations in blood serum, especially in cows with clinical mastitis.

## Figures and Tables

**Table 1:** The blood serum metabolic parameters in the cows with subclinical and clinical forms of mastitis and healthy cows.

Metabolic Parameter	Clinical Mastitis (1)	Subclinical Mastitis (2)	Healthy (3)	ANOVA (*p* Values)
Glucose (mmol/L)	2.0 ± 0.33	1.91 ± 0.34	2.03 ± 0.48	0.649
NEFAs (mmol/L)	0.59 ± 0.19 ^a^	0.44 ± 0.17 ^b^	0.34 ± 0.10 ^c^	0.001
BHB (mmol/L)	0.58 ± 0.34 ^a^	0.35 ± 0.16 ^b^	0.31 ± 0.09 ^b^	0.004
Triglycerides (mmol/L)	0.23 ± 0.16 ^a^	0.51 ± 0.34 ^b^	0.70 ± 0.29 ^c^	0.001
Total protein (g/L)	75.00 ± 5.15 ^a^	66.10 ± 10.23 ^b^	59.46 ± 6.64 ^c^	0.001
Albumin (g/L)	25.29 ± 4.20 ^a^	27.35 ± 3.54 ^a,b^	29.50 ± 3.03 ^b^	0.002
Globulin (g/L)	49.58 ± 7.53 ^a^	38.74 ± 8.93 ^b^	29.69 ± 4.74 ^c^	0.001
Urea (mmol/L)	6.41 ± 2.20 ^a^	4.23 ± 0.96 ^b^	5.35 ± 1.24 ^c^	0.001
Creatinine (mmol/L)	49.16 ± 25.82 ^a^	45.74 ± 16.44 ^a^	72,91 ± 13.70 ^b^	0.001
Total bilirubin (mmol/L)	7.29 ± 3.21 ^a^	2.63 ± 1.67 ^b^	3.31 ± 2.90 ^b^	0.001
AST (I/U)	102.90 ± 56.20 ^a^	76.76 ± 40.27 ^b^	66.53 ± 14.17 ^b^	0.018
ALT (I/U)	20.76 ± 8.74 ^a^	27.04 ± 6.85 ^b^	36.79 ± 8.94 ^c^	0.001
ALP (I/U)	64.60 ± 36.81 ^a^	39.56 ± 14.21 ^b^	35.34 ± 9.06 ^b^	0.001
GGT (I/U)	28.61 ± 7.48 ^a^	28.06 ± 11.44 ^a^	18.49 ± 7.01 ^b^	0.001
LDH (I/U)	1736.91 ± 401.37 ^a^	1066.05 ± 203.69 ^b^	984.68 ± 152.14 ^b^	0.001
Ca (mmol/L)	1.95 ± 0.24	2.13 ± 0.33	2.02 ± 0.36	0.181
P (mmol/L)	2.91 ± 2.86	2.53 ± 0.71	2.38 ± 0.47	0.615
Mg (mmol/L)	1.01 ± 0.30 ^a^	0.77 ± 0.33 ^b^	0.66 ± 0.35 ^b^	0.003

Different small Latin letters (^a^, ^b^, ^c^) indicate statistically significant differences between groups.

**Table 2:** Milk composition in clinical form of mastitis (Group 1), subclinical form of mastitis (Group 2), and healthy cows (Group 3).

	Clinical Mastitis (1)	Subclinical Mastitis (2)	Healthy Cows (3)	ANOVA (*p* Values)
Milk fat (%)	5.76 ± 4.23 ^a^	5.89 ± 2.85 ^a^	2.04 ± 1.04 ^b^	0.001
Lactose (%)	3.51± 0.82 ^a^	4.16 ± 0.47 ^b^	4.52 ± 0.32 ^b^	0.001
Total protein in milk (%)	3.72 ± 0.62 ^a^	3.45 ± 0.44 ^a^	3.01 ± 0.36 ^b^	0.001
Casein (%)	2.27 ± 0.34 ^a^	2.31 ± 0.34 ^a^	2.66 ± 0.32 ^b^	0.001
BHB (mmol/L)	0.27 ± 0.33 ^a^	0.22 ± 0.18 ^a^	0.04 ± 0.01 ^b^	0.004
Acetone (mmol/L)	0.22 ± 0.12	0.17 ± 0.15	0.19 ± 0.12	0.655
Urea (MUN) (mg/dL)	28.84 ± 15.12	24.72 ± 10.46	16.45 ± 8.92	0.089
LDH (I/U)	1898.86 ± 348.10 ^a^	1287.90 ± 750.21 ^b^	913.47 ± 92.1 ^c^	0.001
CFUs (*1000/mL)	765.78 ± 1223.02 ^a^	33.85 ± 50.07 ^b^	26.16 ± 33.01 ^b^	0.002
SCC (*1000/mL)	7933.5 ± 2944 ^a^	2957.5 ± 397.8 ^b^	586.41 ± 120.25 ^c^	0.001

Different small Latin letters (^a^, ^b^, ^c^) indicate statistically significant differences between groups. * indicates the value (CFU, SCC) in the table is increased 1000 times

**Table 3:** The Pearson correlation coefficients of metabolic parameters in blood serum and parameters of milk composition (bolded values denote statistically significant correlations, *p* < 0.05).

	Milk Fat	Total Protein	Lactose	SCC	CFUs	Casein	Urea	BHB	Acetone	LDH
Glucose	−0.143	−0.036	−0.051	−0.041	0.155	−0.164	−0.172	0.080	0.197	−0.032
NEFAs	**0.552**	0.072	**−0.317**	**0.268**	**0.299**	0.154	**0.489**	0.211	−0.154	**0.392**
BHB	**0.382**	0.098	−0.129	**0.438**	0.094	**0.263**	**0.308**	0.200	−0.243	**0.446**
Triglycerides	**−0.325**	−0.240	**0.326**	**−0.262**	−0.234	−0.202	−0.198	−0.163	−0.024	**−0.298**
Total protein	0.166	0.228	**−0.422**	**0.370**	**0.276**	0.091	0.081	**0.280**	0.127	**0.443**
Albumin	−0.087	**−0.297**	**0.328**	**−0.266**	**−0.449**	−0.224	−0.016	−0.205	0.008	**−0.278**
Globulin	0.176	**0.337**	**−0.526**	**0.450**	**0.416**	0.183	0.093	**0.336**	0.162	**0.492**
Urea	0.134	0.008	−0.078	**0.299**	−0.002	0.095	0.228	−0.054	**−0.258**	0.224
Total bilirubin	**0.385**	**0.295**	**−0.313**	**0.385**	0.239	0.107	**0.252**	**0.284**	0.159	**0.321**
AST	0.152	**0.252**	−0.201	**0.427**	0.170	0.185	0.158	0.239	−0.063	0.233
ALT	**−0.292**	**−0.293**	**0.280**	−0.193	**−0.438**	**−0.341**	−0.208	−0.200	0.085	**−0.36**
ALP	−0.091	0.109	0.023	0.192	0.017	0.078	−0.011	−0.020	0.028	0.190
GGT	0.225	0.171	−0.135	**0.322**	0.059	0.189	0.142	0.127	−0.086	0.230
LDH	0.220	**0.499**	**−0.466**	**0.604**	0.203	**0.361**	0.140	**0.263**	0.065	**0.674**
Ca	−0.079	−0.173	0.122	−0.110	**−0.376**	−0.106	0.003	**−0.262**	−0.108	0.010
P	−0.132	−0.117	−0.108	0.204	−0.108	−0.090	0.037	−0.056	0.184	0.157
Mg	0.040	**0.260**	−0.226	0.182	−0.034	0.248	0.012	0.003	0.009	**0.360**

## Data Availability

The data used to support the findings of this study are available in the present manuscript.

## Supplementary Materials

The following are available online at https://www.mdpi.com/article/10.3390/pathogens12111349/s1, Table S1: One-way ANOVA of blood serum biochemical parameters and milk composition parameters. Table S2. PCA results—blood serum biochemical parameters, Table S3. PCA results—milk composition parameters.
