# Supplementary material for: Assessment of Mastitis Patterns in Serbian Dairy Cows: Blood Serum Metabolic Profile and Milk Composition Parameters
Source: Pathogens. 2023 Nov 14;12(11):1349. doi: 10.3390/pathogens12111349 (PMC10674276; doi:10.3390/pathogens12111349)
Supplement: Supplementary file 1 [file pathogens-12-01349-s001.zip › pathogens-2628403-supplementary.pdf]

Table S1. One-way ANOVA of blood serum biochemical parameters and milk composition parameters.

Dependent Variable	Test of SS Whole Model vs. SS Residual										
	Multiple R	Multiple R^2	Adjusted R^2	SS Model	df Model	MS Model	SS Residual	df Residual	MS Residual	F	p
Total protein	0.657282	0.432019	0.412766	2562	2	1281	3369	59	57	22.43840	0.000000
Albumin	0.435304	0.189489	0.162014	185	2	93	792	59	13	6.89680	0.002034
Globulin	0.753312	0.567479	0.552817	4162	2	2081	3172	59	54	38.70475	0.000000
Total bilirubin	0.653856	0.427528	0.408122	268	2	134	358	59	6	22.03089	0.000000
AST	0.355478	0.126364	0.096750	15032	2	7516	103923	59	1761	4.26694	0.018588
GGT	0.467406	0.218468	0.191976	1287	2	643	4604	59	78	8.24639	0.000695
Glucose	0.120537	0.014529	-0.018877	0	2	0	9	59	0	0.43493	0.649368
Ca	0.237345	0.056333	0.024344	0	2	0	6	59	0	1.76101	0.180784
P	0.127723	0.016313	-0.017032	3	2	2	194	59	3	0.48922	0.615569
Urea	0.498419	0.248422	0.222945	50	2	25	153	59	3	9.75074	0.000219
Triglyceride	0.602465	0.362964	0.341370	2	2	1	4	59	0	16.80823	0.000002
BHB	0.477489	0.227996	0.201826	1	2	0	3	59	0	8.71221	0.000484
Mg	0.419342	0.175848	0.147910	1	2	1	6	59	0	6.29436	0.003328
ALP	0.484907	0.235135	0.209208	10801	2	5401	35135	59	596	9.06891	0.000368
ALT	0.633106	0.400823	0.380512	2682	2	1341	4009	59	68	19.73420	0.000000
Creatinine	0.519922	0.270319	0.245584	8588	2	4294	23183	59	393	10.92865	0.000092
LDH	0.779785	0.608065	0.594779	7368028	2	3684014	4749141	59	80494	45.76761	0.000000
NEFA	0.546396	0.298549	0.274771	1	2	0	2	59	0	12.55566	0.000029
Milk fat (%)	0.497129	0.247137	0.221616	189	2	94	575	59	10	9.68375	0.000231
Milk protein (%)	0.509335	0.259422	0.234318	5	2	3	14	59	0	10.33375	0.000142
Lactose (%)	0.590661	0.348881	0.326809	11	2	6	21	59	0	15.80659	0.000003
SCC (*1000/mL)	0.644543	0.415436	0.395620	656947106	2	328473553	924395825	59	15667726	20.96498	0.000000
CFU (CFU*1000/mL)	0.438073	0.191908	0.164515	7830882	2	3915441	32974477	59	558889	7.00575	0.001862
Casein	0.472489	0.223245	0.196915	2	2	1	6	59	0	8.47853	0.000580
Urea (mg/dl)	0.280555	0.078711	0.047481	1627	2	813	19041	59	323	2.52035	0.089059
BHB (mmol/l)	0.408835	0.167146	0.138914	1	2	0	3	59	0	5.92038	0.004537
Acetone (mmol/l)	0.119219	0.014213	-0.019203	0	2	0	2	59	0	0.42533	0.655539
LDH (IU/L)	0.660746	0.436585	0.417486	10472805	2	5236402	13515211	59	229071	22.85926	0.000000

Table S2. PCA results – blood serum biochemical parameters.

a) PCA Loadings.

Variable	Factor Loadings (Varimax normalized) Extraction: Principal components	
	Factor 1	Factor 2
Total protein	0.591474	-0.536983
Albumin	-0.052621	0.721027
Globulin	0.560219	-0.678787
Total bilirubin	0.605705	-0.247423
AST	0.665653	0.219627
GGT	0.522233	0.008121
Glucose	-0.140780	-0.287939

Ca	-0.036026	0.203117
P	-0.001395	-0.187055
Urea	0.724719	0.431964
Triglyceride	-0.449751	0.264523
BHB	0.780976	0.126675
Mg	0.367781	-0.065564
ALP	0.361263	-0.319133
ALT	-0.255037	0.714829
Creatinine	-0.030606	0.516182
LDH	0.701030	-0.210673
NEFA	0.644636	-0.248346
Expl. Var	4.346446	2.799897
Prp. Totl	0.241469	0.155550

b) Eigenvalues.

Value	Eigenvalues; Extraction: Principal components			
	Eigenvalue	% Total variance	Cumulative Eigenvalue	Cumulative %
1	4.880449	27.11360	4.880449	27.11360
2	2.265895	12.58830	7.146343	39.70191

c) PCA scores.

Case	Factor Scores; Rotation: Varimax normalized; Extraction: Principal components			
	Factor 1	Factor 2 - Clinical	Factor 2- Subclinical	Factor 2- Healthy
1	0.11716	-2.33033		
2	0.16863	-0.20569		
3	0.56202	-1.44194		
4	0.05045	-1.36273		
5	-0.16992	-1.49105		
6	0.47760	-0.82948		
7	-0.14839	-0.71128		
8	0.90815	-0.31669		
9	0.34187	-1.63744		
10	-0.17806	-2.82698		
11	1.90116	-0.29190		
12	1.54412	-0.42443		
13	1.49317	0.20380		
14	2.01304	0.73855		
15	1.17281	0.28178		
16	2.46361	1.20504		
17	3.12501	2.14334		
18	0.72303	-1.46503		
19	1.06070	-0.46384		

20	1.24090	-0.54091		
21	1.02980	-0.87190		
22	1.63043	-0.17970		
23	1.05438	-0.45569		
24	-0.27462		-0.74608	
25	-0.34208		0.20898	
26	-0.83589		-0.09316	
27	0.53472		0.33632	
28	-0.07542		0.74193	
29	-0.62734		-0.53911	
30	-0.94728		-0.30050	
31	-1.27264		-0.92496	
32	0.26967		-0.63318	
33	-0.04166		0.50956	
34	-1.10664		-0.29568	
35	-0.50175		0.47001	
36	-0.65321		-0.87175	
37	0.42657		1.01663	
38	-0.50802		0.44348	
39	-0.47291		-0.31600	
40	-0.67642		-0.12949	
41	-0.88774		-0.82156	
42	-1.34525		-1.03841	
43	0.68777		0.47086	
44	-0.98460			0.20487
45	-0.91445			0.87125
46	-0.95123			0.87448
47	-0.98825			1.37779
48	-0.51105			1.39058
49	-1.09052			-0.45237
50	-0.64894			1.00763
51	-0.18020			0.56458
52	-0.18537			2.29503
53	-0.81932			0.39243
54	-0.70057			0.97426
55	-0.37987			0.61244
56	-0.59261			0.73034
57	-0.56342			0.52722
58	-0.65921			0.52444
59	-0.99803			0.43412
60	-1.04262			1.54875
61	-0.94819			1.08036
62	-0.77309			0.82845

Table S3. PCA results – milk composition parameters.

a) PCA loadings.

	PCA loadings		
Variable	Category value	Component 1	Component 2
Milk fat (%)		0.505820	0.639217
Milk protein (%)		0.703483	0.076944
Lactose (%)		-0.759569	0.345575
SCC (*1000/mL)		0.684450	-0.250515
CFU (CFU*1000/mL)		0.564019	-0.417851
Casein		0.476658	0.602306
Urea (mg/dl)		0.323144	0.653459
BHB (mmol/l)		0.656735	-0.289406
Acetone (mmol/l)		0.222583	-0.610712
LDH (IU/L)		0.673746	0.060271
Group {Clinical mastitis}	1	0.771779	-0.194688
Group {Subclinical mastitis}	2	-0.061064	0.548596
Group {Healthy}	3	-0.746765	-0.352268

b) Eigenvalues.

	Principal Components Analysis Eigenvalues			
Component	Eigenvalues	% Total variance	Cumulative eigenvalue	Cumulative %
1	4.537710	34.90546	4.537710	34.90546
2	2.484391	19.11070	7.022101	54.01616

c) PCA scores.

	Scores	
	Component 1	Component 2
1	1.46414	-0.34514
2	0.30796	0.48298
3	-1.24428	-1.12743
4	1.62115	-1.87718
5	1.25712	-2.17232
6	4.78921	-1.00896
7	2.54269	-0.58541
8	2.64595	-2.60884
9	3.97430	-3.19097
10	4.24618	-3.31851
11	1.29574	2.20331
12	1.21916	0.39009
13	0.89607	-0.66562
14	1.64667	0.92032
15	4.99866	-1.96909

16	1.46853	2.47929
17	2.52341	1.19844
18	3.05029	3.39264
19	2.63753	3.06646
20	0.50940	-0.63449
21	0.81596	-0.28327
22	3.97373	-2.37412
23	2.25736	-1.14751
24	-1.13709	1.00287
25	0.05829	0.70966
26	-1.00257	1.07978
27	-0.46351	1.39271
28	-1.21376	0.90690
29	-0.55323	0.86729
30	-0.35252	1.99174
31	-1.32018	0.89403
32	0.49674	1.27413
33	0.69387	-0.23623
34	0.50663	3.25410
35	1.25179	2.37414
36	1.03569	1.44782
37	-0.15588	1.97843
38	-0.26597	0.38496
39	-0.39455	2.13771
40	-0.06428	1.63956
41	-0.85887	0.89454
42	-0.21320	-0.26676
43	0.05709	0.98621
44	-1.97548	-0.05129
45	-2.55343	-0.73241
46	-2.58305	-1.31105
47	-1.61464	-0.36660
48	-2.11798	-0.61803
49	-2.67345	-0.80849
50	-1.86473	-0.23285
51	-2.81860	-1.83368
52	-1.82908	-2.61075
53	-2.62151	-0.80658
54	-2.44149	-1.65284
55	-2.64113	-1.42254
56	-2.88779	-1.22842
57	-2.41559	-0.27256
58	-1.93984	0.08483
59	-2.07570	0.03031

60	-2.76793	-0.82476
61	-2.94438	-0.86825
62	-2.23562	-0.01229
